# Structure Identification and In Vitro Anticancer Activity of Lathyrol-3-phenylacetate-5,15-diacetate

**DOI:** 10.3390/molecules22091412

**Published:** 2017-08-25

**Authors:** Jian-ye Zhang, Wen-jing Huang, Hong-mei Sun, Yun Liu, Xiao-qin Zhao, Si-li Tang, Ming-na Sun, Sheng Wang, Jia-jun Li, Ling-ling Zhang, Jun-hua Zhou, Qian-rong Pan, Hu-biao Chen

**Affiliations:** 1 Key Laboratory of Molecular and Clinical Pharmacology, School of Pharmaceutical Sciences and the Fifth Affiliated Hospital, Guangzhou Medical University, Guangzhou 511436, China; short_j@sina.com (W.-j.H.); liokvie@163.com (Y.L.); tangsilisixin@126.com (S.-l.T.); ming--na85@163.com (M.-n.S.); wsheng0108@163.com (S.W.); rizyazyun@163.com (J.-j.L.); splm88@163.com (L.-l.Z.); xhua0@163.com (J.-h.Z.); panqr2017@163.com (Q.-r.P.); 2 Infinitus (China) Company Ltd., Jiangmen 529156, China; wendy.sun@infinitus-int.com; 3 Pediatric Department, Guangdong Women and Children Hospital and Health Institute, Guangzhou 511400, China; zhaoxiaoqinde@126.com; 4 School of Chinese Medicine, Hong Kong Baptist University, Hong Kong, China

**Keywords:** natural products, Caper Euphorbia Seed, lathyrol-3-phenylacetate-5,15-diacetate, diterpenoids, lung cancer, apoptosis

## Abstract

Natural products from the genus *Euphorbia* show attention-attracting activities, such as anticancer activity. In this article, classical isolation and structure identification were used in a study on Caper Euphorbia Seed. Subsequently, MTT and wound healing assays, flow cytometry, western blotting, Hoechst 33258 staining and fluorescence microscopy examination were applied to investigate the anticancer activity of the obtained compounds. In a result, lathyrol-3-phenyl- acetate-5,15-diacetate (deoxy Euphorbia factor L1, DEFL1) was isolated from Caper Euphorbia Seed. Moreover, the NMR signals were totally assigned. DEFL1 showed potent inhibition against lung cancer A549 cells, with an IC_50_ value of 17.51 ± 0.85 μM. Furthermore, DEFL1 suppressed wound healing of A549 cells in a concentration-dependent manner. Mechanically, DEFL1 induced apoptosis, with involvement of an increase of reactive oxygen species (ROS), decrease of mitochondrial membrane potential (ΔΨm), release of cytochrome *c*, activity raise of caspase-9 and 3. Characteristic features of apoptosis were observed by fluorescence microscopy. In summary, DEFL1 inhibited growth and induced apoptosis in lung cancer A549 cells via a mitochondrial pathway.

## 1. Introduction

Cancer has become the increasing public health problem and the major threat to human health for its high morbidity and mortality [1,2,3]. Under current understanding, chemotherapy is one of the key treatment methods, which has achieved great therapeutic success for certain malignant tumors [4,5,6]. However, some aspects are limiting factors of chemotherapy, including side effects and resistance, particularly multi-drug resistance (MDR) [7,8,9]. Thus, developing novel drugs with better efficacy to treat cancer is the effective strategy [10,11,12].

Natural resources are still the key provider of novel drugs despite the development of combinatorial chemistry, which can synthesize thousands of compounds quickly [13,14]. *Euphorbia* is the largest genus of the spurge family, containing more than 2000 species, some of which have been applied as medicinal plants to treat various diseases, including cancer [15]. Caper Euphorbia Seed, used as a Traditional Chinese Medicine is the seeds of *Euphorbia lathyris* L. [16]. The main constituents of Caper Euphorbia Seed are lathyrane diterpenoids [17], and a series of diterpenes L1–L8 based on the lathyrane skeleton have been isolated from the seeds. The biological activities of these lathyrane-type diterpenes have been studied, showing cytotoxicity to cancer cells and the ability to reverse MDR [18,19].

As part of ongoing research, we have exhaustively investigated Caper Euphorbia Seed, examining the chemical isolation and structure identification of its components and their pharmacological aspects [20,21,22,23]. During these studies, lathyrol-3-phenylacetate-5,15-diacetate (deoxy Euphorbia factor L1, DEFL1) was isolated from Caper Euphorbia Seed. In this article we report its structure identification and total NMR signal assignment. Furthermore, DEFL1 was shown to inhibit the growth of and induce apoptosis in A549 cells via a mitochondrial pathway.

## 2. Results

### 2.1. Structure Identification and NMR Signal Assignments

**Figure 1:**
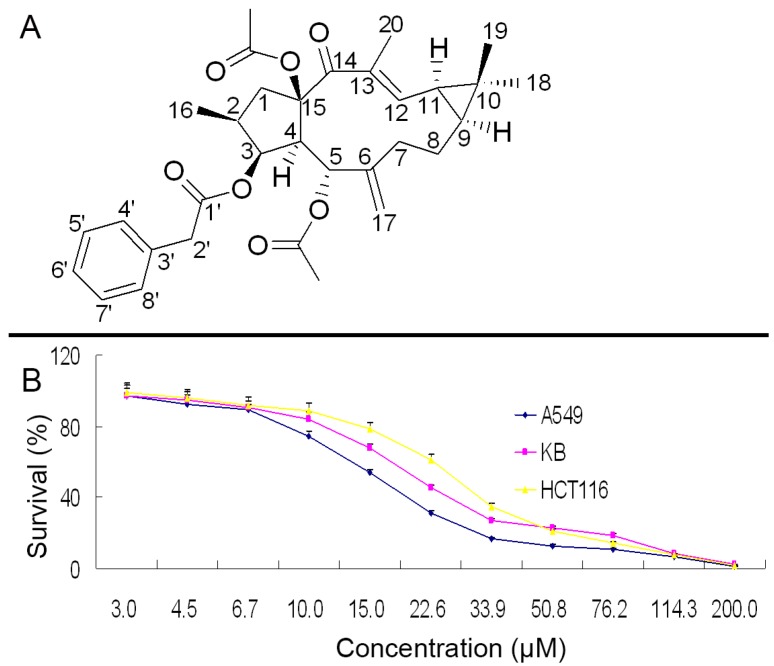
(**A**) Chemical structure of lathyrol-3-phenylacetate-5,15-diacetate (deoxy Euphorbia factor L1, DEFL1); (**B**) Growth inhibition curve of DEFL1 against A549, KB and HCT116 cells.

Lathyrol-3-phenylacetate-5,15-diacetate (deoxy Euphorbia factor L1, DEFL1) was obtained as white flaky crystals. Colorless crystals were obtained from dichloromethane-petroleum ether. The molecular formula C_32_H_40_O_7_ was determined by high resolution mass spectrometry (HRMS) showing *m*/*z* 537.2848 (M + H), which indicates 13 degrees of unsaturation. The ^1^H-NMR spectrum displayed five benzene protons with chemical shifts between 7.15 and 7.50. Moreover, 30 carbon signals were visible in the ^13^C-NMR data. The information supplied by the molecular formula, ^1^H-NMR and ^13^C-NMR implied that DEFL1 contains one monosubstituted benzene ring. The ^13^C-NMR spectrum also showed four carbonyls at 196.8, 171.3, 170.7 and 169.8 ppm. The DEPT spectrum indicated that DEFL1 has six methyl carbons, five methylene carbons, ten methine carbons, and nine quaternary carbons. The ^1^H-, ^13^C-NMR and heteronuclear multiple bond correlation (HMBC) data listed in Table 1, together with the data in reference [19] allowed the full assignment of the 1D NMR signals and the determination of the structure as shown in Figure 1A.

### 2.2. Growth Inhibition in A549, KB and HCT116 Cells

The growth suppression results of DEFL1 were determined by an MTT assay to give IC_50_ values of 17.51 ± 0.85, 24.07 ± 1.06, and 27.18 ± 1.21 μM for A549, KB and HCT116 cells, respectively (Figure 1B). Among these three cancer cell lines, A549 cells showed the most sensitivity (*p* < 0.05), thus A549 cells were selected for further investigation.

### 2.3. Suppressed Wound Healing in A549 Cells

**Figure 2:**
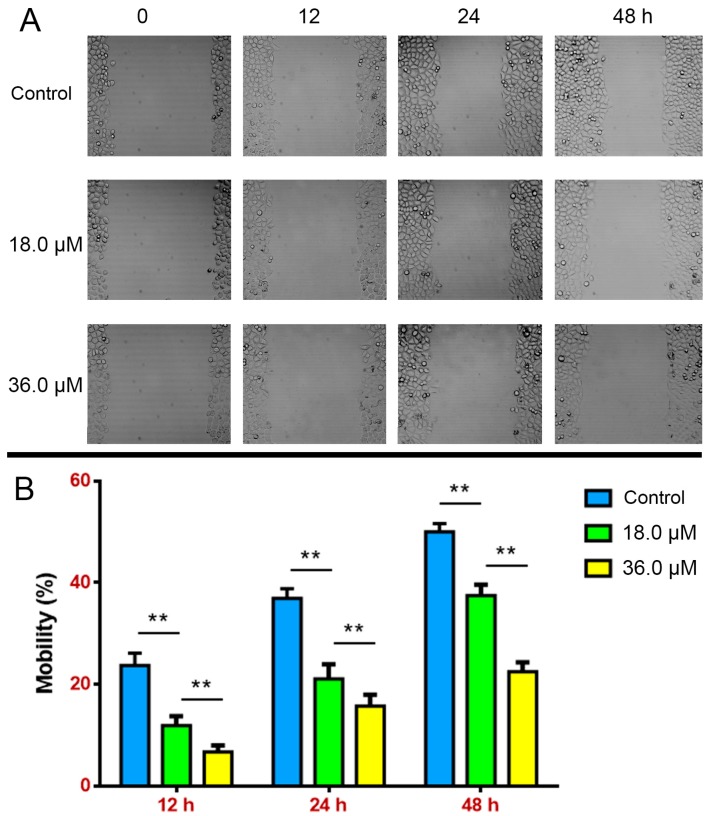
DEFL1 inhibition of A549 cells growth and migration. (**A**) Microscopic observation of A549 cells wound healing results after treatment of different concentrations for the indicated time; (**B**) Statistical analysis results of different concentration groups compared at 12, 24 and 48 h, respectively.** *p* < 0.01.

To further determine the growth inhibition of DEFL1, a wound healing assay was performed, which could also measure cell migration ability [4]. After A549 cells were treated with the indicated concentrations of DEFL1 for 12, 24, 48 h, the percentage of wound healing was determined (Figure 2). At 12 h, the healing percentages were 23.72 ± 2.21%, 11.86 ± 1.72% and 6.70 ± 1.14% for control, 18.0 and 36.0 μM DEFL1, respectively. At 24 h, the healing percentages were 36.89 ± 1.78%, 21.09 ± 2.58% and 15.68 ± 2.09% for control, 18.0 and 36.0 μM DEFL1, respectively. At 48 h, the healing percentages were 50.02 ± 1.49%, 37.47 ± 1.98% and 22.48 ± 1.70% for control, 18.0 and 36.0 μM DEFL1, respectively. These results implied that DEFL1 suppressed the proliferation and migration of A549 cells.

### 2.4. Increase of Intracellular ROS Levels in A549 Cells

**Figure 3:**
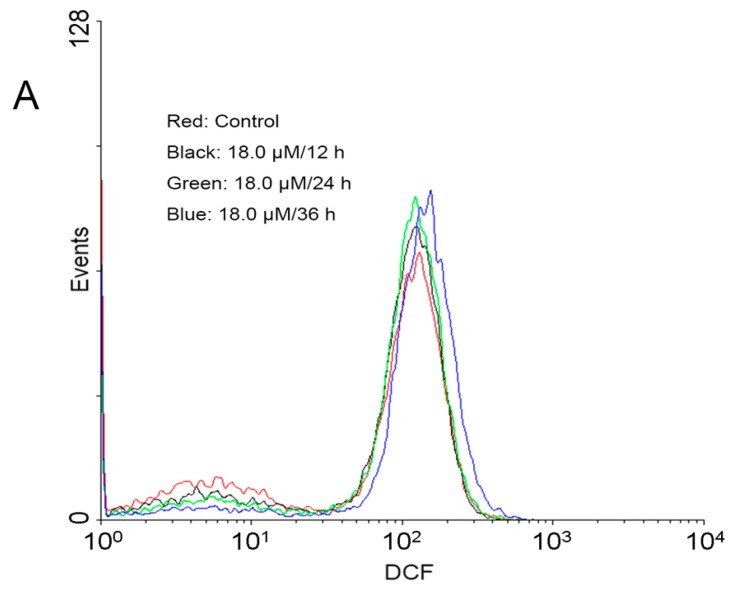

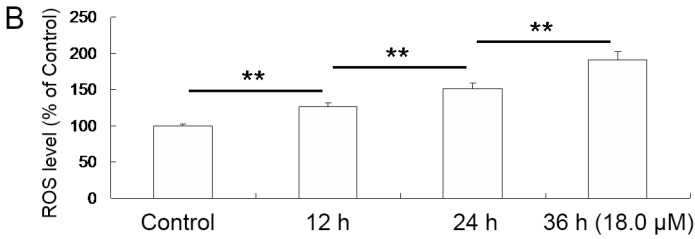
DEFL1 treatment led to an increase of ROS levels in A549 cells in a time-course manner. (**A**) ROS generation increase was observed after exposured to 18.0 μM DEFL1 for the indicated time; (**B**) Intracellular ROS levels of A549 cells is expressed as a percentage of control. ** *p* < 0.01.

According to current knowledge, a raise of intracellular ROS levels causes apoptosis [20]. To verify this, generation of ROS was measured with DCFH-DA, which can be hydrolyzed and oxidized to DCF, a fluorescent dye that can be detected by flow cytometry [21]. After A549 cells were treated with 18.0 μM DEFL1 for 12, 24 and 36 h, the intracellular ROS levels were 126.66 ± 4.46%, 151.15 ± 7.09% and 191.04 ± 11.56% of control, respectively (Figure 3).

### 2.5. Reduced Mitochondrial Membrane Potential (ΔΨm) of A549 Cells

**Figure 4:**
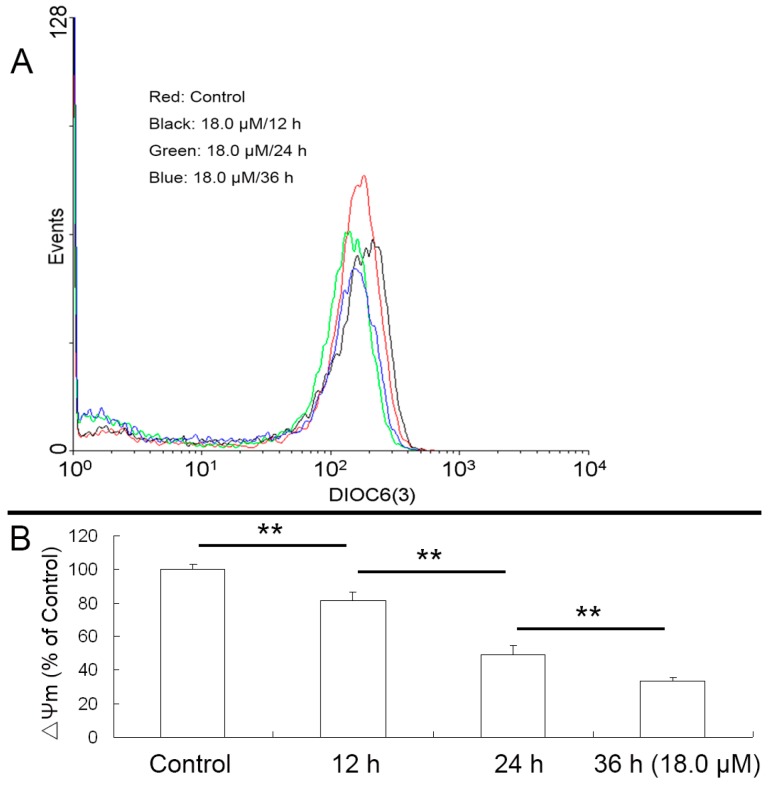
Decrease of ΔΨm was observed after A549 cells were exposed to DEFL1 showing a time-dependent pattern. (**A**) Reduction of ΔΨm measured by flow cytometry; (**B**) A549 cells ΔΨm at different time was expressed as a percentage of control. ** *p* < 0.01.

It is well known that an increase of ROS levels can cause the loss of ΔΨm, which plays a crucial role in the mitochondrial pathway [21]. Indeed, decreases of ΔΨm in a time-dependant manner were observed (Figure 4). After treatment with 18.0 μM DEFL1, the levels of ΔΨm were 81.42 ± 4.98%, 49.03 ± 5.33% and 33.37 ± 2.19% of control after 12, 24 and 36 h, respectively.

### 2.6. Release of Cytochrome C

**Figure 5:**
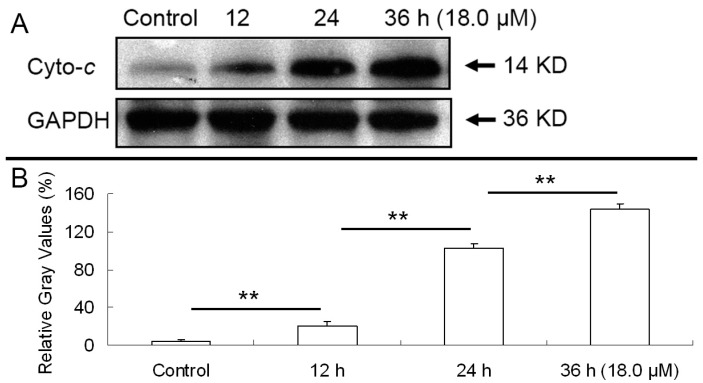
Exposure to DEFL1 resulted in release of cytochrome *c*. (**A**) Western blotting results of cytochrome *c* after various time of treatment; (**B**) Relative gray values of western blotting results were determined by Image J. ** *p* < 0.01.

Functional mitochondria depend on the maintenance of mitochondrial membrane potential (ΔΨm), loss of which results in release of cytochrome *c* [21]. To confirm this, cytosolic cytochrome *c* was detected by western blot analysis. After A549 cells were treated with 18.0 μM DEFL1 for different time, the release of cytochrome *c* was determined (Figure 5). Relative gray values of cytochrome *c* were 3.94 ± 2.17%, 20.16 ± 4.47%, 102.10 ± 4.90%, 144.09 ± 4.89%, for control, 12, 24, 36 h, respectively.

### 2.7. Increased Activities of Caspase-9, -3 and Apoptosis Confirmation by Hoechst 33258 Staining

**Figure 6:**
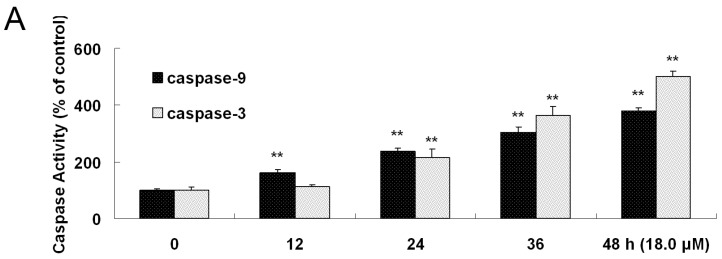

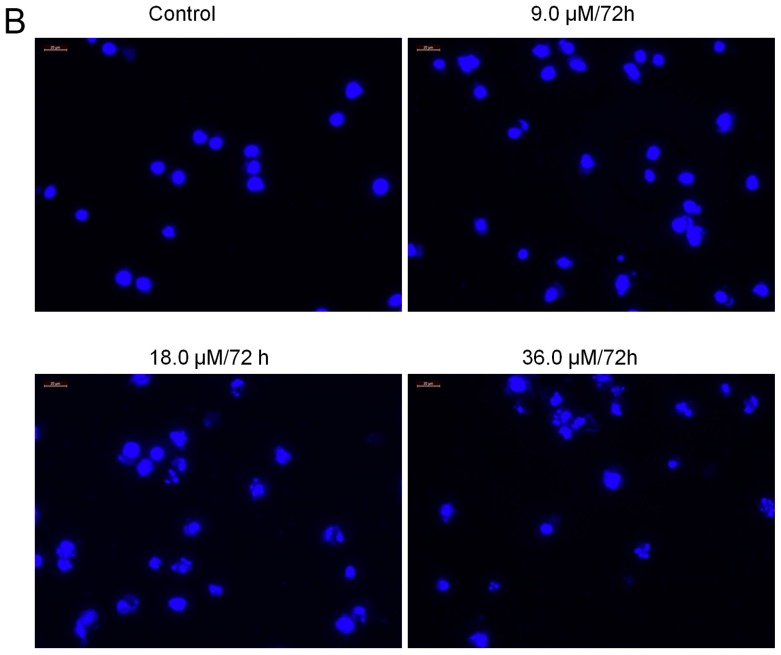
Caspase-9, -3 were activated and apoptosis character of Hoechst 33258 staining was observed. (**A**) Activities of caspase-9 and -3 in A549 cells were significantly enhanced by DEFL1 in time-dependent manner; (**B**) Hoechst 33258 staining confirmed the apoptosis, displaying morphological changes of apoptosis. ** *p* < 0.01.

It is widely known that cytosolic cytochrome *c* can activate procaspase-9. Subsequently, caspase-9 activates downstream caspases, including caspase-3 as apoptosis executer [21]. After A549 cells were exposed to 18.0 μM DEFL1 for indicated time, the activities of caspase-9 and -3 were measured by a caspase colorimetric assay kit following the manufacturer’s protocol (Figure 6A). Caspase-9 activity was 160.19 ± 12.31%, 237.76 ± 8.92%, 305.14 ± 18.34%, 380.06 ± 9.98% of control, 12, 24, 36, 48 h, respectively. Caspase-3 activity was 112.50 ± 6.82%, 215.97 ± 29.86%, 364.43 ± 31.98%, 500.56 ± 19.01% of control, 12, 24, 36, 48 h, respectively. Furthermore, the apoptotic cells were measured by fluorescence microscopy showing shrinkage of cell volume, and fragmentation of the nuclei (Figure 6B).

## 3. Discussion

Among different kinds of cancers, lung cancer remains the most common cancer throughout the world, regarding new cases and death for its high rates of morbidity and mortality [24,25]. Furthermore, non-small cell lung cancer (NSCLC) is the most common form of lung cancer [26,27]. Cancer chemotherapy offers effective treatment and developing novel anticancer drugs is a promising strategy to overcome cancer [28,29]. More and more evidence suggests that the success of anti-tumor chemotherapy depends on the discovery and development of novel drugs [23,30].

Despite the advent of combinatorial chemistry which can generate thousands of new chemicals rapidly, natural products remain the key source for developing novel drugs [31,32,33]. The genus *Euphorbia* provides various types of compounds including sesquiterpenoids, diterpenoids, triterpenoids, steroids, phenolics and flavonoids, among which, diterpenoids are the vital type for the biological activity [15,34]. Considerable attention has been focused on macrocyclic diterpenoids derived from lathyranes with a 5/11/3-membered ring that have exhibited anticancer and MDR- reversing activities [35,36].

In this paper we report the isolation, structure identification and apoptosis induction in NSCLC A549 cells of lathyrol-3-phenyl- acetate-5,15-diacetate (deoxy Euphorbia factor L1, DEFL1) showed strong growth inhibition in A549 cells with an IC_50_ value of 17.51 ± 0.85 μM (Figure 1B). What’s more, DEFL1 suppressed cell growth and migration in experiments of wound healing of A549 cells in a dose-dependent manner (Figure 2). To elucidate the anticancer mechanism, apoptosis induced by DEFL1 was investigated.

Apoptosis is a highly regulated death process deciding homeostasis and development of multicellular organisms [37,38,39]. It is characterized by a series of well-organized processes including activation of the family of cysteinyl aspartate-specific proteinases (caspase), reduction of cell volume, membrane blebbing, internucleosomal DNA cleavage, compaction of cytoplasmic organelles and fragmentation of nuclear chromatin [40,41,42]. What’s more, apoptosis is one of the important mechanisms of action of anticancer agents [43,44]. The mitochondrial pathway and death receptor pathway are two major apoptosis pathways that depend on different apoptotic stimuli [45,46]. Mitochondria have been suggested to play a critical role in the regulation of apoptosis, which is related to the generation of ROS and the disruption of redox homeostasis [47,48,49]. Indeed, an increase of ROS levels was observed in A549 cells after treatment with DEFL1 (Figure 3). Mitochondrial dysfunction, including permeability transition, loss of mitochondrial potential (ΔΨm) and release of cytochrome *c* from mitochondria into cytosol can lead to apoptosis [50,51]. In our research, the loss of ΔΨm (Figure 4) and release of cytochrome *c* (Figure 5) were detected in DEFL1- treated A549 cells.

**Figure 7:**
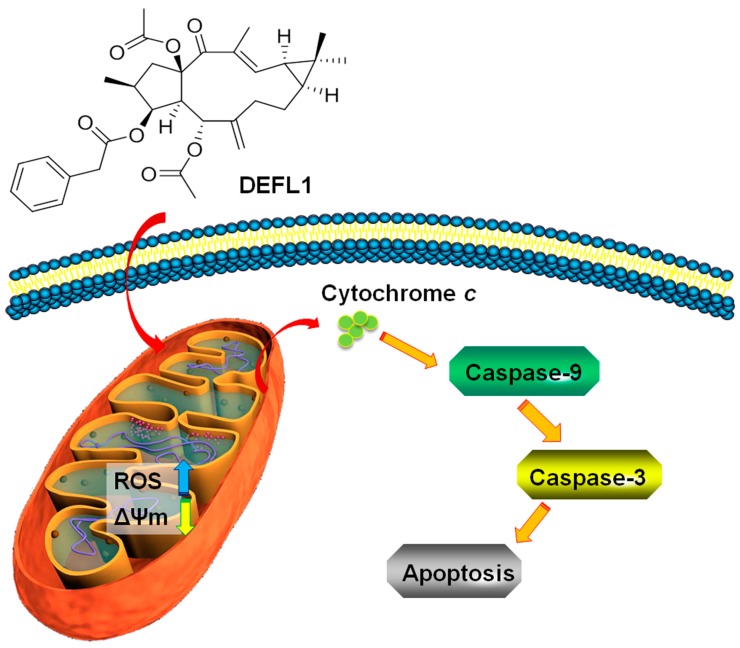
Proposed mechanism of DEFL1 regarding apoptosis inducement in A549 cells.

As far as the mitochondrial pathway is concerned, caspase-9 is activated after cytochrome *c* is released to cytosol [52,53]. Subsequently, complex formation of cytochrome *c*, apoptotic protease activation factor 1 (Apaf-1) and procaspase-9 can activate the caspase-9, which brings up the activation of downstream caspases, including casapse-3 [54,55,56]. Consistently, an activity increase of caspase-9 and -3 was recorded (Figure 6A). Furthermore, Hoechst 33258 staining provided more evidence showing apoptosis of reduction of cell volume and fragmentation of nuclear chromatin (Figure 6B). Our results implied that DEFL1 induced apoptosis of A549 cells via a mitochondrial pathway and the related mechanisms are summarized in Figure 7.

## 4. Materials and Methods

### 4.1. General Procedures

3-(4,5-Dimethylthiazolyl-2)-2,5-diphenyltetrazolium bromide (MTT) was obtained from MP Biomedicals Inc. (Santa Ana, CA, USA). RPMI 1640 was purchased from Thermo Fisher Scientific Inc. (Waltham, MA, USA). Fetal bovine serum (FBS) were bought from Zhejiang Tianhang Biotechnology Co., Ltd. (Hangzhou, China). Anti-GAPDH antibodies were from Bioworld Technology Inc. (St. Louis Park, MN, USA), while anti-cytochrome c antibodies were acquired from Cell Signalling Technology Co. (Danvers, MA, USA). The caspase activity assay kit was a product of Beyotime Co. (Shanghai, China). Other routine laboratory reagents were obtained from commercial sources in analytical or HPLC grade. NMR data were recorded on a Inova-500 NB spectrometer (Varian, Palo Alto, CA, USA). CDCl_3_ was used as solvent and TMS as internal standard. Chemical shifts (δ) are expressed in ppm with reference to the TMS peak. Mass spectra were acquired on an ultrahigh liquid chromatography instrument coupled with a quadrupole time-of-flight mass spectrometer (G6540A, UPLC-QTOF-MS, Agilent Technologies, Santa Clara, CA, USA). IR spectra were obtained on a 5DX-FTIR spectrophotometer (Nicolet, Madison, WI, USA). Column chromatography was performed on silica gel (200–300 mesh, Qingdao Marine Chemical, Qingdao, China). Fractions were monitored by TLC visualized by heating silica gel plates sprayed with 5% H_2_SO_4_ in EtOH.

### 4.2. Plants, Extraction, Isolation and Characterization of DEFL1

Caper Euphorbia Seed (seeds of *Euphorbia lathyris* L.) was purchased from Anguo, Hebei Province and identified by Professor Hubiao Chen (School of Chinese Medicine, Hong Kong Baptist University). A voucher specimen is deposited at the Herbarium of the School of Pharmaceutical Sciences, Guangzhou Medical University. Powder of Caper Euphorbia Seed (16 kg) was refluxed with 95% EtOH (4 L/kg, 3 h/per time, three times) to obtain the ethanolic extract. Subsequently, the extract was concentrated and suspended in H_2_O and partitioned successively with cyclohexane (4 L/per time, three times), EtOAc (4 L/per time, three times) and *n*-BuOH (4 L/per time, three times) to afford the corresponding extracts. The EtOAc extract was separated and repeatedly purified by silical gel (petroleum ether-ethyl acetate) and Sephadex LH-20 column chromatography (methylene chloride-methanol) to get lathyrol-3-phenyl- acetate-5,15-diacetate (deoxy Euphorbia factor L1, DEFL1): 40 mg; mp 126–128 °C; IR (KBr) *v*_max_ 1742, 1729, 1648, 1623, 1265, 1237, 1129, 1010, 1006 cm^−1^; *m*/*z* 537.2848 (M + H)^+^; NMR: see Table 1.

### 4.3. Cell Lines and Culture

A549, KB and HCT116 cells provided by Prof. Li-Wu Fu (Cancer Center, Sun Yat-sen University) were cultivated in RPMI 1640 medium containing 100 U/mL penicillin, 100 μg/mL streptomycin and 10% FBS in an incubator with a humidified atmosphere of 5% CO_2_ at 37 °C [57]. Mycoplasma contamination was determined every two months regularly.

### 4.4. Cell Viability Assay

A549 cells were plated in 96-well plates and allowed to adhere for 24 h. Cells were then incubated with varying concentrations of indicated compounds or fractions. After 68 h, MTT was added to each well and plates were then incubated for another 4 h. After that, formazan crystals were dissolved with 200 μL DMSO and the absorbance at 540 nm was measured by a microplate reader with 655 nm as reference wavelength. Finally, cell survival was calculated as: survival (%) = (mean experimental absorbance/mean control absorbance) × 100% [58].

### 4.5. Wound Healing Assay

To perform the wound healing experiments, A549 cells were seeded in the six-well plates and cells were cultured to reach confluence overnight. Then, the 200 μL pipette tip was applied to scratch the monolayer cell to build the wound healing model. After that, the wounded cell layer was washed to discard loose cells. Medium containing different concentrations of DEFL1 was added to the plates. After indicated time of culture, images were captured. Results of cell growth and motility were examined based on the percentage of the healing area [4].

### 4.6. Measurement of ROS Generation

Briefly, DCF fluorescence intensity is directly related to the amount of ROS produced by the cells. After A549 cells were treated with DEFL1 for 24 h, 5 × 10^5^ cells were harvested, washed with ice-cold PBS and incubated with DCFH-DA (50 μM of the final concentration) in the dark at 37 °C for 20 min. Subsequently, cells were washed twice with ice-cold PBS and resuspended in 1 mL PBS. ROS generation quantity was examined based on 10,000 cells each sample by a FACS Caliber flow cytometer (Beckman Coulter, Brea, CA, USA) at 488 nm of the excitation wavelength and 530 nm of the emission wavelength. The data were analyzed by CellQuest software (BD Biosciences Inc., Franklin Lakes, NJ, USA) and expressed as MFI [59].

### 4.7. Determination of Mitochondrial Potential (ΔΨm)

A549 cells at density of 5 × 10^5^ cells/mL were treated with 18.0 µM DEFL1 for indicated time to determine ΔΨm. Thereafter, the cells were collected, centrifuged at 600 g for 5 min and then washed with ice-cold PBS. Subsequently, cells were incubated with 40 nM DiOC6 (3) for 20 min in the dark at 37 °C. After that, stained cells were washed twice with ice-cold PBS once before being resuspened in 1 mL PBS. Finally, the quantity of DiOC6 (3) maintained 10,000 cells each sample was determined by a FACS Caliber flow cytometer (Beckman Coulter, Brea, CA, USA) at 484 nm of excitation wavelength and 501 nm of emission wavelength. The recorded data were analyzed by CellQuest software (BD Biosciences Inc., Franklin Lakes, NJ, USA) and expressed as a mean fluorescence intensity (MFI) [60].

### 4.8. Subcellular Isolation for Western Blot Analysis of Cytosolic Cytochrome c

The indicated extract buffer contained 250 mM sucrose, 20 mM Hepes-KOH (pH 7.5), 10 mM KCl, 1.5 mM MgCl_2_, 1 mM DTT, 1 mM EGTA, 1 mM EDTA, 0.1 mM PMSF and 0.02 mM aprotinin. After treatment with DEFL1, A549 cells were harvested and resuspended in 5-fold volume of ice-cold extract buffer for 40 min at 4 °C. Subsequently, A549 cells were centrifuged at 1200× *g* for 10 min at 4 °C and the supernatant was then centrifuged at 12,000× *g* for 15 min at 4 °C to give the final supernatant as cytosolic fraction. Subsequently, the final supernatant after reduplicative centrifugation was collected and dissolved in 5 × loading buffer (250 mM Tris-Cl of pH 6.8, 50% glycerol, 10% sodium dodecyl sulphate, 1.25‰ bromphenol blue and 0.5 M dithiothreitol). After that the samples were heated for 15 min at 100 °C. Then the samples were subjected to western blotting analysis and cytochrome *c* protein was examined by anti-cytochrome *c* antibody in the ratio of 1:1000 [59].

### 4.9. Measurement of Caspase-9 and -3 Activities

The activities of caspase-9 and -3 were determined by a caspase colorimetric assay kit following the manufacturer’s protocol. To be brief, 1 × 10^6^ A549 cells were exposed to 18.0 μM DEFL1 for 12, 24, 36 and 48 h, respectively. Then, cells were collected, washed twice with ice-cold PBS and lysed with the buffer. After that, the lysates were detected for protease activity by the caspase-specific peptide conjugated with color reporter molecule *p*-nitroanaline. Generally, the caspase enzymatic activities in the given cell lysates were proportional to the color reaction. Finally, the chromophore *p*-nitroanaline cleaved by caspases was measured by spectrophotometry at 405 nm [20].

### 4.10. Hoechst 33258 Staining

After exposure to DEFL1, both floating and trypsinized adherent A549 cells were collected, washed once with ice-cold PBS and fixed with 1 mL 4% paraformaldehyde for 20 min. Subsequently, cells were washed once with ice-cold PBS. Then the cells were incubated in 1 mL PBS containing 10 μM Hoechst 33258 for 30 min at 37 °C, washed twice. Finally, the results were observed under a fluorescence microscope equipped with standard excitation filters (Leica Dmirb, Wetzlar, Germany) in random microscopic fields at 400 × magnification [54].

### 4.11. Statistical Analysis

Results were analyzed with t-test or one-way ANOVA with the SPSS software (13.0, SPSS Inc., Chicago, IL, USA). Data were expressed as means ± SD of at least triplicate determinations. * *p* < 0.05 and ** *p* < 0.01 were indicative of significance.

## 5. Conclusions

In summary, DEFL1 inhibits growth and induces apoptosis in A549 cells with involvement of the mitochondrial pathway (Figure 7), characterized by an increase of ROS levels, decrease of mitochondrial potential (ΔΨm), release of cytochrome *c*, activation of caspase-9 and -3 and observation of typical apoptosis features after Hoechst 33258 staining.

## Figures and Tables

**Table 1:** ^1^H- and ^13^C-NMR data of DEFL1 (δ, CDCl_3_, 500 and 125 MHz for ^13^C and ^1^H, respectively).

Position		^13^C	^1^H	HMBC (H→C)
1		48.3	3.40 (1H, dd, *J* = 8, 14 Hz) 1.44 (1H, *J* = 14, 11 Hz)	2, 3, 4, 14, 15
2		37.4	2.01 (1H, m)	1, 3, 4, 15, 16
3		80.6	5.60 (1H, t, *J* = 3 Hz)	1, 1’, 15
4		52.2	2.79 (1H, dd, *J* = 3.6, 10 Hz)	5, 6, 14
5		65.9	6.12 (1H, d, *J* = 10.4 Hz)	4, 6, 7, 15, 5-CO
6		144.4		
7		35.0	2.11 (1H, m), 2.20 (1H, m)	5, 6, 8, 9, 17
8		21.7	2.01 (1H, m), 1.71 (1H, m)	6, 9
9		35.3	1.1 (1H , m)	18, 19
10		25.2		
11		29.0	1.40 (1H, dd, *J* = 8.4, 11.6 Hz)	9, 10, 12, 13
12		146.7	6.54 (1H, d, 11.2 Hz)	9, 14, 20
13		134.1		
14		196.8		
15		92.3		
16		13.7	0.73 (3H, d, *J* = 6.4 Hz)	1, 2, 3
17		115.6	5.01 (1H, s), 4.74 (1H, s)	5, 6, 7
18		28.4	1.19 (3H, s)	9, 10, 11, 19
19		16.8	1.18 (3H, s)	9, 10, 11, 18
20		12.4	1.70 (3H, s)	12, 13, 14
5-COCH_3_	CH_3_	21.3	1.96 (3H, s)	5-CO
	CO	170.7		
15-COCH_3_	CH_3_	22.0	2.20 (3H, s)	15-CO
	CO	169.8		
3-OPhAc	1′	171.3		
	2’	41.6	3.64 (1H, d, *J* = 15 Hz), 3.62 (1H, d, *J* = 15 Hz)	1′, 3′, 4′, 8′
	3′	134.0		
	6′	127.1	7.25 (1H, m)	4′, 8′
	4′, 8′	129.5	7.31 (2H, m)	2’, 3′
	5′, 7′	128.5	7.33 (2H, m)	3′
